# A Study of Intratumoral Fibrous Interstitium in the Growth and Proliferation Process in Rectal Carcinoid Tumors

**DOI:** 10.1155/DTE.5.197

**Published:** 1999

**Authors:** Shuji Matsumura

**Affiliations:** Third Department of Internal Medicine Ohashi Hospital Toho University School of Medicine 2-17-6 Ohashi, Meguro-ku Tokyo 153-8515 Japan

## Abstract

The aim of the study was to investigate the relationship between the fibrous interstitium found in the spaces between tumor nests and the growth and proliferation process in 33 rectal carcinoid lesions (26 resected endoscopically and 7 resected surgically).

The proportion of the tumor occupied by fibrous interstitium (F/T ratio) was measured in tissue specimens using an image analyzer, and the composition and distribution pattern of the fibrous interstitium were classified based on 21 lesions in which special staining was performed successfully. The results were then compared with tumor nest malignancy and preoperative endoscopic ultrasonography (EUS) findings for 23 lsions.

The F/T ratio was significantly higher in the 16 lesions smaller than 5 mm and composed of low malignancy tumor nests (0.49 ±  0.14) than in the 17 lesions 5 mm or larger (0.25 ± 0.10). However, the F/T ratio was high (mean 0.41) for 2 of the 17 lesions 5 mm or larger comprised of highly malignant tumor nests.

The fibrous interstitium was composed of part of the smooth muscle resulting from destruction of the muscularis mucosae and collagenous fibers. The 9 lesions with mixed interstitium in which smooth muscle predominated were comprised of low malignancy tumor nests with a significantly higher F/T ratio (0.55 ±  0.09). In contrast, of the 12 lesions with separated interstitium, the 4 lesions in which collagenous fibers predominated had a significantly higher F/T ratio (0.34 ± 0.05) than the 8 lesions in which smooth muscle predominated (0.18 ±  0.05). Three of these 4 lesions had infiltrated as far as the deeper sections of the submucosal layer on EUS, and 1 of the lesions was a highly malignant tumor with an invasion depth of adventitia.

The EUS internal echo images of the tumors were classifiable into: (1) homogenous low-echoic; (2) honeycomb; (3) multiple high-echoic dots; and (4) unvisualized. These classifications reflected the tumor structure (structure of the dominant nest, F/T ratio, and fibrous interstitium distribution pattern) and depth of invasion.

At the immature stage, there were large amounts of destroyed muscularis mucosae present in the spaces between the dispersed tumor nests. At the mature stage, the tumor nests formed a single solid mass, and in lesions that had infiltrated to the deeper sections of the submucosal layer or were highly malignant, the number of collagenous fibers inside the tumor increased, suggesting that fibrous interstitium is closely related to rectal carcinoid tumor growth and proliferation. These histologic findings can be predicted from EUS, and may therefore be useful in assisting preoperative diagnosis.


*Diagnostic and Therapeutic Endoscopy*, Vol. 5, pp. 197–210
Reprints available directly from the publisher
Photocopying permitted by license only

© 1999 OPA (Overseas Publishers Association) N.V.
Published by license under
the Harwood Academic Publishers imprint,
part of The Gordon and Breach Publishing Group.
Printed in Malaysia.

### A Study of Intratumoral Fibrous Interstitium in the Growth and Proliferation Process in Rectal Carcinoid Tumors

SHUJI MATSUMURA*

*Third Department of Internal Medicine, Ohashi Hospital, Toho University School of Medicine, 2-17-6 Ohashi, Meguro-ku, Tokyo 153-8515, Japan*

*(Received 29 June 1998; In final form 9 November 1998)*

**The aim of the study was to investigate the relationship between the fibrous interstitium found in the spaces between tumor nests and the growth and proliferation process in 33 rectal carcinoid lesions (26 resected endoscopically and 7 resected surgically).**

**The proportion of the tumor occupied by fibrous interstitium (F/T ratio) was measured in tissue specimens using an image analyzer, and the composition and distribution pattern of the fibrous interstitium were classified based on 21 lesions in which special staining was performed successfully. The results were then compared with tumor nest malignancy and preoperative endoscopic ultrasonography (EUS) findings for 23 lesions.**

**The F/T ratio was significantly higher in the 16 lesions smaller than 5 mm and composed of low malignancy tumor nests ($0.49 \pm 0.14$) than in the 17 lesions 5 mm or larger ($0.25 \pm 0.10$). However, the F/T ratio was high (mean 0.41) for 2 of the 17 lesions 5 mm or larger comprised of highly malignant tumor nests.**

**The fibrous interstitium was composed of part of the smooth muscle resulting from destruction of the muscularis mucosae and collagenous fibers. The 9 lesions with mixed interstitium in which smooth muscle predominated were comprised of low malignancy tumor nests with a significantly higher F/T ratio ($0.55 \pm 0.09$). In contrast, of the 12 lesions with separated interstitium, the 4 lesions in which collagenous fibers predominated had a significantly higher F/T ratio ($0.34 \pm 0.05$) than the 8 lesions in which smooth muscle predominated ($0.18 \pm 0.05$). Three of these 4 lesions had infiltrated as far as the deeper sections of the submucosal layer on EUS, and 1 of the lesions was a highly malignant tumor with an invasion depth of adventitia.**

**The EUS internal echo images of the tumors were classifiable into: (1) homogenous low-echoic; (2) honeycomb; (3) multiple high-echoic dots; and (4) unvisualized. These classifications reflected the tumor structure (structure of the dominant nest, F/T ratio, and fibrous interstitium distribution pattern) and depth of invasion.**

**At the immature stage, there were large amounts of destroyed muscularis mucosae present in the spaces between the dispersed tumor nests. At the mature stage, the tumor nests formed a single solid mass, and in lesions that had infiltrated to the deeper sections of the submucosal layer or were highly malignant, the number of collagenous fibers inside the tumor increased,**

* Tel.: 03-3468-1251. Fax: 03-3468-1269.

**suggesting that fibrous interstitium is closely related to rectal carcinoid tumor growth and proliferation. These histologic findings can be predicted from EUS, and may therefore be useful in assisting preoperative diagnosis.**

*Keywords:* Endoscopic ultrasonography, Fibrous interstitium, Image analysis, Rectal carcinoid

### INTRODUCTION

The detectability of tumorous lesions of the colon has increased dramatically in recent years, thanks to improvement in colonoscopes and diagnostic techniques. Such is also the case with rectal carcinoids. Haraguchi *et al.* [1] reviewed 496 cases of rectal carcinoid tumor from Japanese literature in 1988. These were initially thought to be slow-growing tumors in which tumor size was closely related to the degree of malignancy, [1,2] but recent reports of malignant tumors smaller than 10 mm [3–5] indicate the need for a more accurate indicator of malignancy than tumor size. The aim of the present study was to examine the usefulness of the fibrous interstitium found in the spaces between tumor nests of resected specimens as an indicator of the growth process and histologic degree of malignancy. We also investigated whether it is possible to evaluate clinically fibrous interstitium as an indicator of degree of malignancy before endoscopic or surgical resection, using endoscopic ultrasonography (EUS).

### SUBJECTS

The subjects were 32 patients (33 lesions) with carcinoid tumors of rectal origin who underwent resection and histopathologic testing in 8 years and 2 months between February 1989 and March 1997. The lesions were resected endoscopically in 25 patients (26 lesions) and surgically in 7 patients (7 lesions). Of the 32 patients, 18 were men and 14 were women. Their mean age was 55.1 ± 14.0 years (25–80 years).

The tumor site was Ra (mid part of the rectum) for 8 lesions and Rb (distal part of the rectum) for 25 lesions, as described in *General Rules for Clinical and Pathological Studies on Cancer of the Colon, Rectum and Anus* [6]. Except for 1 lesion with a histopathologic depth of invasion of adventitia treated by abdominoperineal resection, all of the 32 lesions extended as far as the submucosal layer. Endoscopic resection was performed with a high-frequency cautery snare after local injection of saline into the base of the lesion. For 11 of the lesions treated from August 1993, the tumor was resected with a high-frequency cautery snare by first grasping and lifting the tumor with forceps following local injection of saline. The lesion was removed by transanal local resection in 3 patients (3 lesions), transsacral wedge resection in 2 patients (2 lesions), and abdominoperineal resection in 2 patients (2 lesions) (in 1 patient (1 lesion) an advanced rectal carcinoma was resected at the same session).

### METHODS

Endoscopy was performed with an Olympus Optical Co. fiberoptic colonoscope (CF-20I) or electronic colonoscope (CF-200I) to compare macroscopic morphology and the presence or absence of central depressions and ulceration for tumors of differing sizes. Macroscopic morphology was classified as sessile, hemispherical, or semi-pedunculated based on the type of growth.

Material obtained by endoscopic or surgical resection was fixed in 10% formalin and cut through the center of the tumor where possible. After paraffin embedding, variously stained specimens were prepared from consecutive 4- to 5-μm thick sections.

Hematoxylin and Eosin stained (H–E stained) specimens were used to measure the maximum tumor diameter, and to examine the structure of the tumor nests inside the tumor and the proportion of the tumor occupied by the fibrous interstitium found in the spaces between the tumor nests. The tumor structure based on the tumor nests and fibrous interstitium was then compared with the tumor size. The structure of the tumor nests was classified as small solid, trabecular, ribbon-like, nodular and sheet-like based on the criteria by Iwafuchi *et al.* [7] and the nest occupying the greatest area inside the tumor was considered the dominant tumor nest. The proportion of the tumor occupied by the fibrous interstitium was determined by displaying micrographs (×40–100) on a monitor screen linked to a computer and measuring the ratio of the sum total area of fibrous interstitium inside the tumor to the total tumor area (F/T ratio). Each tumor was measured three times using a manually operated image analyzer (Rize Co., type EM), and the mean of the three values was used as the F/T ratio.

The composition of the fibrous interstitium was examined in 21 lesion specimens successfully stained by Azan–Mallory, Elastica van Gieson, and immunohistochemical staining. Primary antibody anti-$\alpha$ smooth muscle actin antibody (×50, 1A4, Immunotech S.A.) was used for immunohistochemical staining. The color was developed with 3,3′ diaminobenzidine (DAB) and was followed by nuclear staining with hematoxylin, using the LSAB (Dako Co., Ltd) method. Cells that had undergone a secondary change were excluded when evaluating positively stained cells.

EUS was performed before treatment with a colonoscope specialized for EUS, Olympus Optical Co. CF-UM3 (12 MHz), or a thin ultrasonic probe UM-2R (12 MHz) or UM-3R (20 MHz) through a working channel of a conventional colonoscope using the water-filling method in both. The maximum tumor diameter, depth of submucosal invasion and internal echo image of the tumor were evaluated. The layer structure of the rectal wall was interpreted using the 5-layer structure described by Aibe [8]. The grade of tumor submucosal invasion was classified into three equal sections in thickness by dividing the high-echoic zone of the third layer, which corresponds to the submucosal layer. Tumors in which the echo extended as far as the second of the three sections (from the lumen side) were classified as superficial types in the submucosal layer, and those in which the tumor extended deeper were classified as deeper types in the submucosal layer. For the 23 lesions in which the histologic depth of invasion was no deeper than the submucosal layer, the accuracy of EUS diagnosis was evaluated comparing the histologic results in the size and depth of the tumor and the intratumoral structure. The CF-UM3 was used for 8 lesions, the UM-2R for 2 lesions, and the UM-3R for 13 lesions.

The Mann–Whitney test was used for the statistical analysis and differences were considered significant if $P < 0.05$.

### RESULTS

### Comparison of Endoscopic Findings and Histologic Tumor Size

The mean histologic tumor size was 5.8 mm, with a range of 1.5–31 mm. Sixteen of the lesions were smaller than 5 mm, sixteen were from 5 mm to less than 10 mm, and one was larger than 10 mm.

The relationship between the endoscopic findings and histologic tumor size for the 32 submucosal lesions was as follows. Of the 16 lesions smaller than 5 mm, 14 (87.5%) were sessile. None of the 16 lesions from 5 mm to less than 10 mm were sessile; 11 (68.8%) were hemispherical and 5 (31.2%) were semipedunculated. None of the lesions had central depressions and the only tumor with ulceration was an 8.0 mm hemispherical lesion (Table I).

The other lesion (31 mm) with a depth of invasion of adventitia was elevated and accompanied by broad based mass with irregular ulceration. The lesion had its base covered with normal peripheral mucosa and was diagnosed as a tumor seated in the submucosal layer.

TABLE I Endoscopic findings and histological tumor size for the submucosal lesions ($n = 32$)

| Histological tumor size (mm) | Endoscopic findings | | |
|---|---|---|---|
| | Sessile | Hemispherical | Semi-pedunculated |
| $< 5$ ($n = 16$) | 14 | 2 | — |
| $\geq 5$ and $< 10$ ($n = 16$) | — | 11* | 5 |

*One of them had surface ulceration.

TABLE II The structure of the dominant nest and histological tumor size ($n = 33$)

| Histological tumor size (mm) | The structure of the dominant nest | | | |
|---|---|---|---|---|
| | Small solid | Trabecular | Ribbon-like | Nodular + Sheet-like |
| $< 5$ ($n = 16$) | 7 | 5 | 4 | — |
| $\geq 5$ and $< 10$ ($n = 16$) | — | — | 15 | 1* |
| $\geq 10$ ($n = 1$) | — | — | — | 1** |

*With surface ulceration endoscopically.
** Advanced case.

### Comparison of the Structure of the Dominant Nest and Histologic Tumor Size

The relationship between the structure of the dominant nest and histologic tumor size was as follows. The 7 lesions with small solid nests and the 5 lesions with trabecular nests were all smaller than 5 mm (12 of 16 lesions: 75.0%). Of the 16 lesions from 5 mm to less than 10 mm, 15 (93.8%) had ribbon-like nests. The lesion with surface ulceration endoscopically and the lesion with a depth of invasion of adventitia had nodular + sheet-like nests (Table II).

### Comparison of F/T Ratio with Histologic Tumor Size and Structure of the Dominant Nest

The relationship between F/T ratio and histologic tumor size was as follows. The mean F/T ratio was 0.49 (0.19–0.67) for the 16 lesions smaller than 5 mm and 0.25 (0.09–0.41) for the 17 lesions 5 mm or larger. The F/T ratio was significantly higher in the lesions smaller than 5 mm than in the lesions 5 mm or larger (Fig. 1).

The relationship between F/T ratio and the structure of the dominant nest was as follows. The mean F/T ratio was 0.58 (0.48–0.65) for the 7 lesions with small solid nests, 0.53 (0.45–0.67) for the 5 lesions with trabecular nests, and 0.24 (0.09–0.41) for the 19 lesions with ribbon-like nests. The F/T ratio was significant higher for the lesions with small solid or trabecular nests than for the lesions with ribbon-like nests. Of the 19 lesions with ribbon-like nests, there was no difference in the F/T ratio for the 4 lesions smaller than 5 mm and the ratio for the 15 lesions 5 mm or larger. The mean F/T ratio for the 2 lesions with nodular + sheet-like nests was high (0.41) (Table III).

### Comparison of the F/T Ratio with Fibrous Interstitium Composition and the Structure of the Dominant Nest

The fibrous interstitium was comprised of collagenous fibers that stained dark blue by Azan–Mallory stain and red by Elastica van Gieson stain, and smooth muscle (part of the muscularis mucosae destroyed by growth of the tumor) darkly stained by immunohistochemical staining. The distribution pattern of the fibrous interstitium in the tumor was broadly classified as mixed (Fig. 2), in which

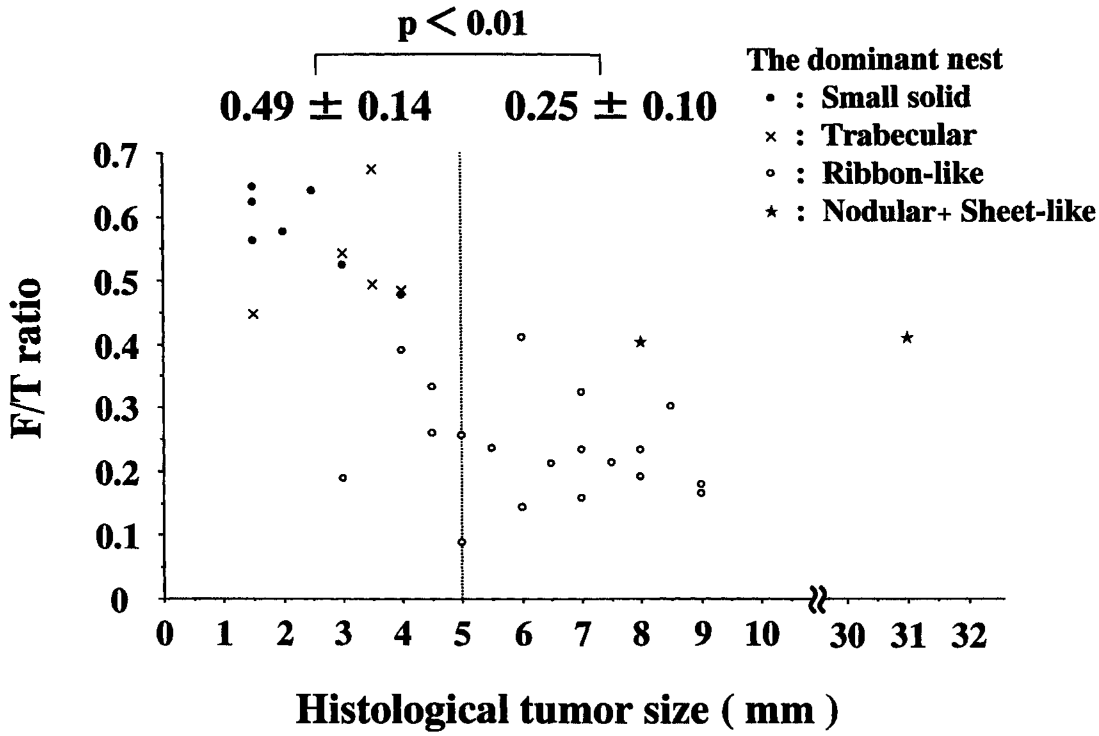

FIGURE 1 F/T ratio, histological tumor size and the structure of the dominant nest.

TABLE III F/T ratio and the structure of the dominant nest ($n = 33$)

| | The structure of the dominant nest | | | |
|---|---|---|---|---|
| | Small solid | Trabecular | Ribbon-like | Nodular + Sheet-like |
| F/T ratio | 0.58 ± 0.06 (7) | 0.53 ± 0.09 (5) | 0.24 ± 0.08 (19) | 0.41* (2) |

Small solid vs. Trabecular: NS; Trabecular vs. Ribbon-like: **; Small solid vs. Ribbon-like: **

Number of lesions in parenthesis.
*Averaged ratio.
**$p < 0.01$.

the smooth muscle and collagenous fibers were mixed together to form a whole, and separated, in which the two types of tissue were separate so that most of the interstitium on the superior side of the tumor was comprised of smooth muscle and the inside of the tumor was comprised of collagenous fibers. In the mixed pattern, smooth muscle constituted over half of the entire interstitium. In the separated pattern, in some lesions most of the interstitium was comprised of the smooth muscle on the superior side of the tumor (Fig. 3), while in others the collagenous fibers inside the tumor were more abundant than the smooth muscle (Fig. 4).

Eight (88.9%) of the 9 lesions with mixed interstitium were comprised of small solid or trabecular nests. The mean F/T ratio (0.55) for these lesions was significantly higher. Of the lesions with a separated pattern, the 8 lesions in which the smooth muscle on the superior side predominated were all comprised of ribbon-like nests, and the

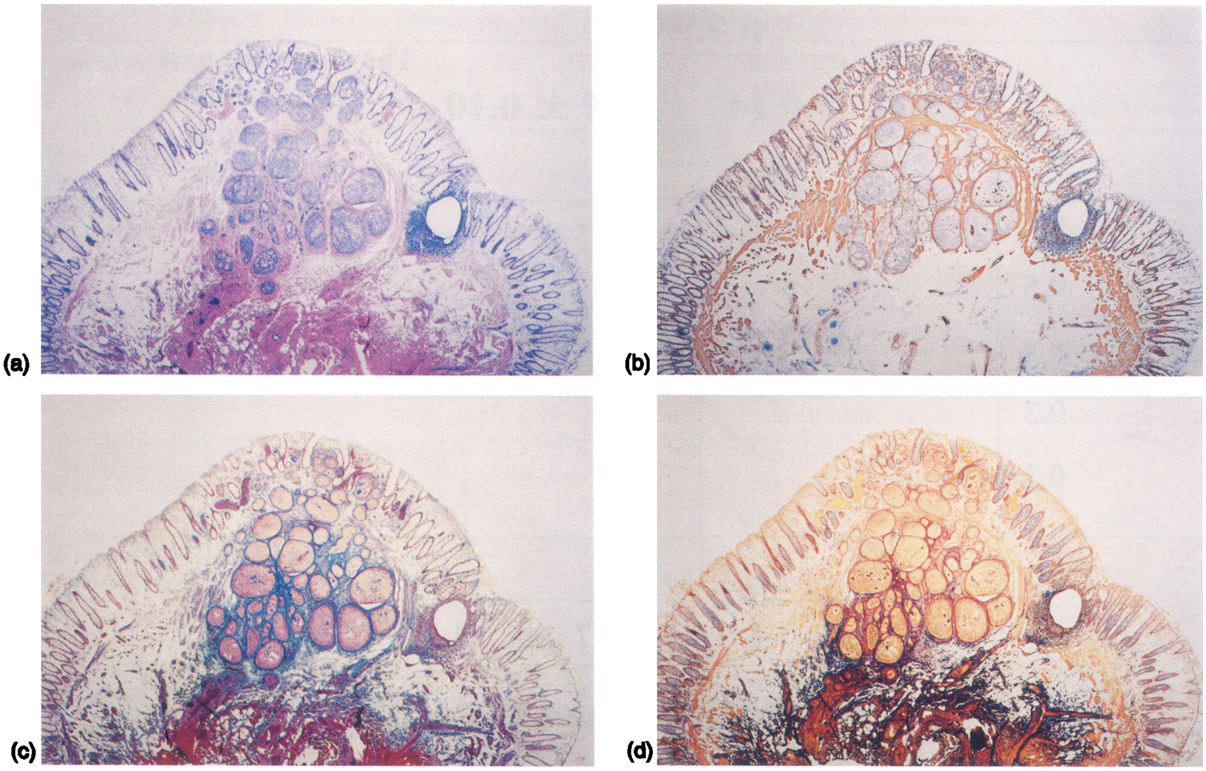

FIGURE 2 The mixed pattern of the fibrous interstitium – mainly smooth muscle. The fibrous interstitium of this carcinoid (tumor size: 2.0 mm, the dominant nest: small solid structure and F/T ratio: 0.58) was comprised of the smooth muscle and the collagenous fibers which were mixed together to form a whole, and the smooth muscle constituted over half of the entire interstitium: (a) stained by H–E stain, (b) anti-smooth muscle actin, (c) Azan-Mallory stain, and (d) Elastica van Gieson stain (× 40).

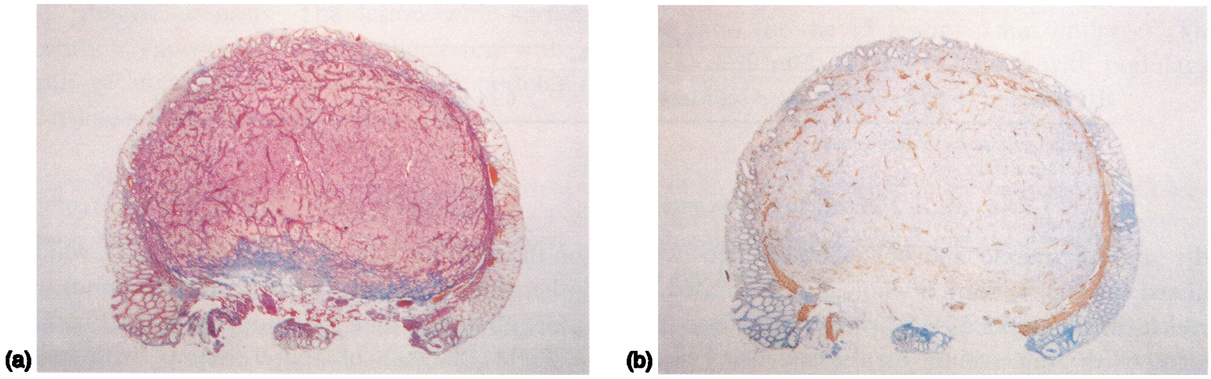

FIGURE 3 The separated pattern of the fibrous interstitium – mainly smooth muscle. The fibrous interstitium of this carcinoid (tumor size: 6.0 mm, the dominant nest: ribbon like structure and F/T ratio: 0.14) was separated on the superior side and the inside of the tumor, and most of the interstitium was comprised of the smooth muscle on the superior side of the tumor: (a) stained by H–E stain and (b) anti-smooth muscle actin (× 12.5).

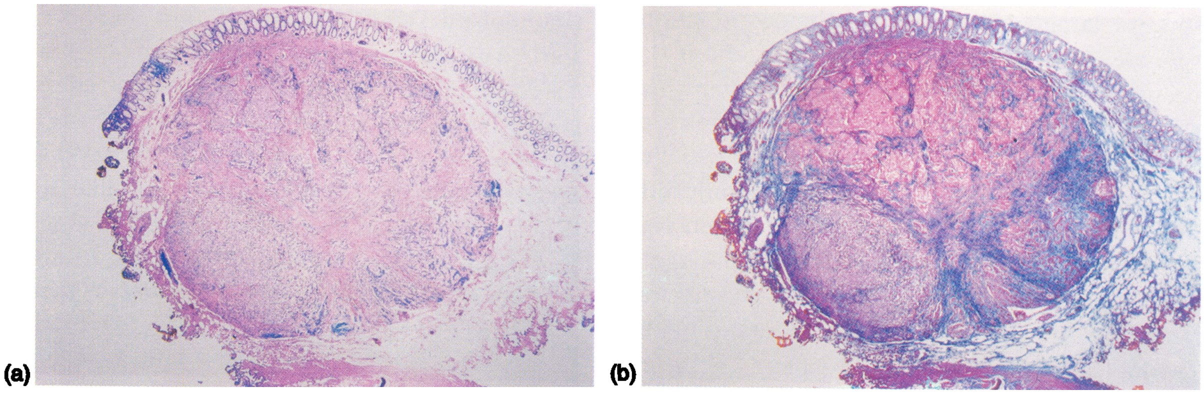

FIGURE 4 The separated pattern of the fibrous interstitium – mainly collagenous fibers. The fibrous interstitium of this carcinoid (tumor size: 7.0 mm, the dominant nest: ribbon like structure and F/T ratio: 0.33) was separated on the superior side and the inside of the tumor, and the collagenous fibers inside the tumor were more abundant than the smooth muscle on the superior side of the tumor: (a) stained by H–E stain and (b) Azan-Mallory stain (× 12.5).

TABLE IV The structure of the dominant nest, the distribution pattern of fibrous interstitium in the tumor and F/T ratio ($n = 21$)

| The structure of the dominant nest | The distribution pattern | | |
|---|---|---|---|
| | Mixed pattern | Separated pattern | |
| | Mainly smooth muscle | Mainly smooth muscle | Mainly collagenous fibers |
| Small solid/Trabecular | 8 | — | — |
| Ribbon-like | 1 | 8 | 3 |
| Nodular + Sheet-like | — | — | 1* |
| F/T ratio | 0.55 ± 0.09 (9) | 0.18 ± 0.05 (8) | 0.34 ± 0.05 (4) |

Significance brackets over the F/T ratio row: ** between 0.55 ± 0.09 (9) and 0.18 ± 0.05 (8); ** between 0.18 ± 0.05 (8) and 0.34 ± 0.05 (4); **[#] between 0.55 ± 0.09 (9) and 0.34 ± 0.05 (4).

Number of lesions in parenthesis.
*Advanced case.
**$p < 0.01$.

F/T ratio (0.18) was significant lower. Three of the 4 lesions in which the collagenous fibers inside the tumor were more abundant than smooth muscle were composed of ribbon-like nests and one was composed of nodular + sheet-like nests. The mean F/T ratio was 0.34, which was significantly higher than the lesions in which the smooth muscle on the superior side predominated (Table IV).

### Comparison of EUS and Histologic Findings

The mean histologic size of the 23 lesions in which EUS was able to be performed before endoscopic or surgical resection was 5.3 mm (1.5–9.0 mm). The mean size for the same lesions on EUS was 6.4 mm (2.5–13 mm), 1.1 mm (17.2%) less than the histologic size.

Of these 23 lesions, 19 (82.6%) were superficial types in the submucosal layer and 4 (17.4%) were deeper submucosal types (Fig. 5). The relationship between the structure of the dominant nest, F/T ratio and depth of submucosal invasion and tumor size on EUS was as follows. All 8 lesions smaller than 5 mm were superficial submucosal lesions. All were composed of small solid or trabecular nests and had a mean F/T ratio of 0.57. Of the 15 lesions

5 mm or larger, 11 superficial submucosal lesions were all comprised of ribbon-like nests and had a mean F/T ratio of 0.20. The 4 deeper submucosal lesions 5 mm or larger had a mean F/T ratio of 0.34. Of the lesions 5 mm or larger, the F/T ratio for the deeper submucosal types was significantly higher than the ratio for the superficial submucosal types. Of the 4 deeper submucosal lesions, the 1 lesion with surface ulceration endoscopically was comprised of nodular + sheet-like nests, and the remaining 3 were comprised of ribbon-like nests. The fibrous interstitium in the latter 3 lesions had a separated distribution pattern and the collagenous fibers inside the tumor was more abundant than the smooth muscle (Table V).

Lesions were broadly divided into homogenous low-echoic lesions and non-homogenous lesions based on the internal echo image of the tumor on EUS. The non-homogenous lesions were further classified into two categories, that is a low-echoic tumor image featuring a honeycomb-like high-echoic pattern and multiple high-echoic dots of varying sizes. The internal echo image of the 4 semipedunculated lesions could not be accurately visualized due to deep echo dumping (Fig. 6).

The relationship between the structure of the dominant nest, F/T ratio and the internal echo image on EUS was as follows. The 8 lesions showing a honeycomb internal echo image (= lesions smaller than 5 mm on EUS) were all formed from small

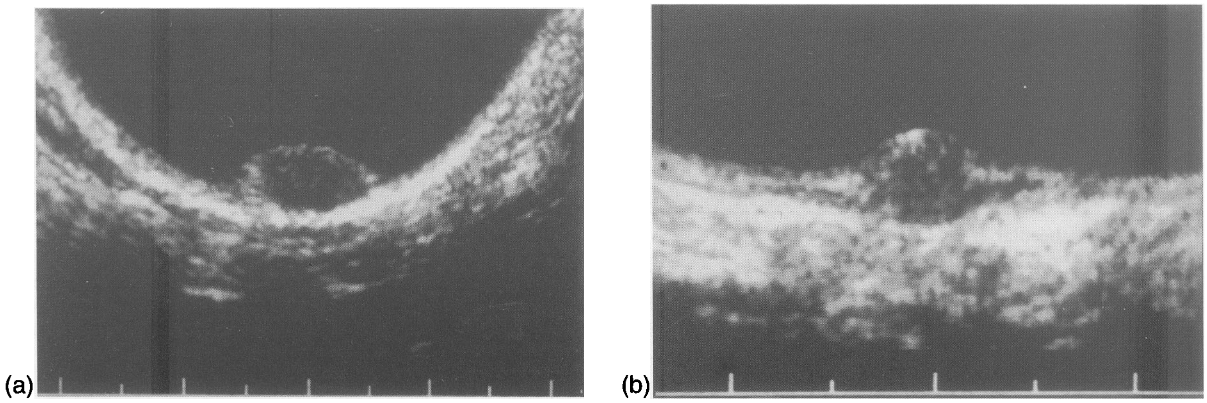

FIGURE 5 The classification of depth of submucosal invasion on EUS: (a) SM-S 12 MHz, 6 cm range and (b) SM-D 12 MHz, 6 cm range.

TABLE V The structure of the dominant nest, F/T ratio and depth of submucosal invasion and tumor size on EUS ($n = 23$)

| The structure of the dominant nest | Depth of submucosal invasion and tumor size (mm) on EUS | | |
|---|---|---|---|
| | SM-S | | SM-D |
| | $<5$ | $\geq 5$ | $\geq 5$ |
| Small solid/Trabecular | 8 | 0 | 0 |
| Ribbon-like | 0 | 11 | 3 |
| Nodular + Sheet-like | 0 | 0 | 1* |
| | ** (vs. next column) | ** (vs. next column) | |
| F/T ratio | 0.57 ± 0.07 (8) | 0.20 ± 0.08 (11) | 0.34 ± 0.05 (4) |

SM-S, superficial submucosal lesions; SM-D, deeper submucosal lesions.
Number of lesions in parenthesis.
*With surface ulceration endoscopically.
**$p < 0.01$.

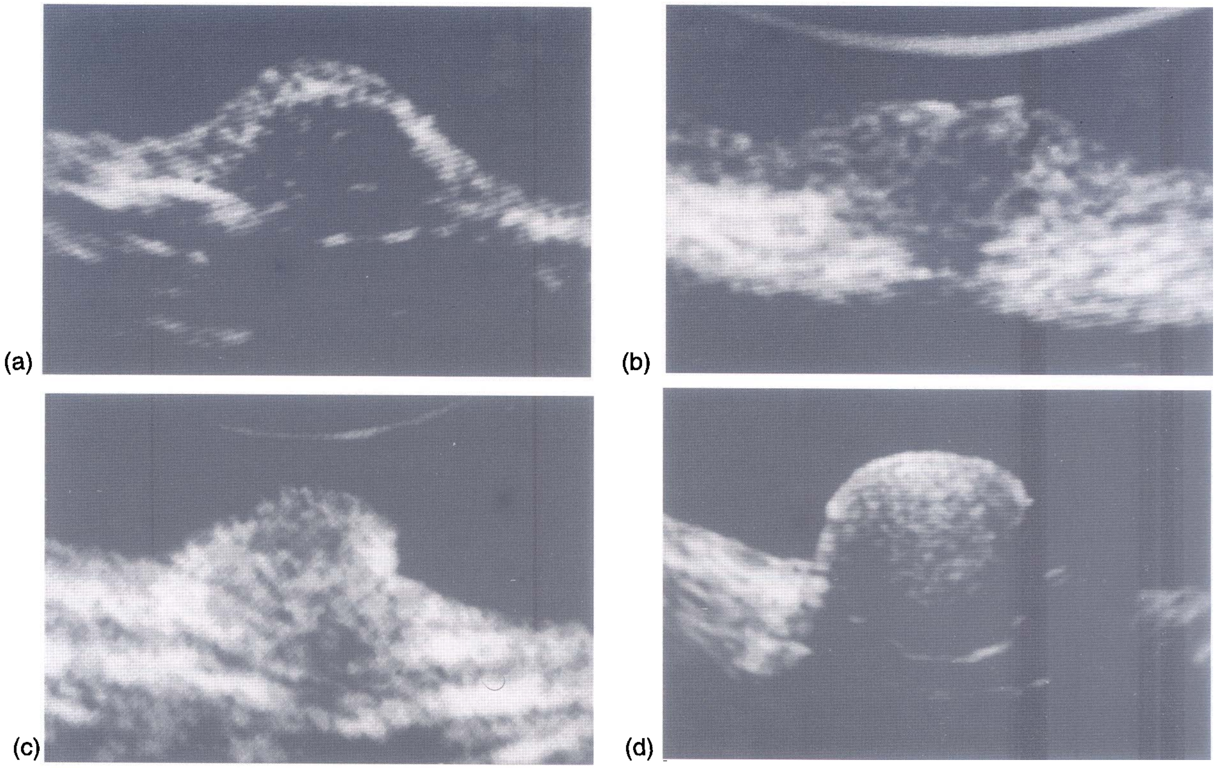

FIGURE 6 The internal echo image on EUS: (a) homogenous low echoic image 20 MHz, 4 cm range), (b) honeycomb image (20 MHz, 2 cm range), (c) multiple high echoic dots image (12 MHz, 6 cm range) and (d) unvisualized because of deep echo dumping (12 MHz, 4 cm range).

solid or trabecular nests and had a mean F/T ratio of 0.57. Of the 5 lesions with an internal echo image characterized by multiple high-echoic dots, 4 were comprised of ribbon-like nests and the 1 lesion with surface ulceration endoscopically was comprised of nodular + sheet-like nests. These lesions had a mean F/T ratio of 0.36. The 6 lesions with a homogenous low-echoic image were composed of ribbon-like nests and had a mean F/T ratio of 0.19. The difference in F/T ratio between the three groups was significant. The 4 unvisualized lesions were composed of ribbon-like nests and had a mean F/T ratio of 0.17, which was not significantly different to the ratio for the lesions exhibiting a homogenous low-echoic image (Table VI).

The relationship between the depth of submucosal invasion and the internal echo image of the tumor on EUS was as follows. Four (80.0%) of the 5 lesions with an internal echo image characterized by the presence of high-echoic dots were deeper submucosal lesions, while those with the other types of internal echo images were all superficial submucosal lesions (Table VII).

### DISCUSSION

Gastrointestinal carcinoid tumors are hardly heteroplastic lesions first described in 1907 by Oberndorfer [9], who coined the term "karzinoide Tumoren" in relation to six slow-growing epithelial tumors of the small intestine resembling ordinary carcinomas but differing in character, which were discovered during autopsy. Carcinoid tumors originating in the rectum were first reported in 1912 by Saltykow [10], who described histologically

TABLE VI The structure of the dominant nest, F/T ratio and the internal echo image on EUS ($n=23$)

| The structure of the dominant nest | The internal echo image | | | |
|---|---|---|---|---|
| | Honeycomb | Multiple high echoic dots | Homogenous low echo | Unvisualized* |
| Small solid/Trabecular | 8** | — | — | — |
| Ribbon-like | — | 4 | 6 | 4 |
| Nodular + Sheet-like | — | 1*** | — | — |
| | | † | | |
| | † | | † | NS |
| F/T ratio | 0.57 ± 0.07 (8) | 0.36 ± 0.05 (5) | 0.19 ± 0.04 (6) | 0.17 ± 0.06 (4) |

Number of lesions in parenthesis.
*Semipedunculated shape endoscopically.
**Smaller than 5 mm on EUS.
***With surface ulceration endoscopically.
†$p < 0.01$.

TABLE VII Depth of submucosal invasion and the internal echo image on EUS ($n=23$)

| Depth of submucosal invasion | The internal echo image | | | |
|---|---|---|---|---|
| | Honeycomb | Multiple high echoic dots | Homogenous low echo | Unvisualized* |
| SM-S | 8 | 1 | 6 | 4 |
| SM-D | — | 4** | — | — |

*Semipedunculated shape endoscopically.
**One of them had surface ulceration endoscopically.

similar rectal tumors detected at autopsy. In 1994 Soga *et al.* [11] studied 2,156 gastrointestinal carcinoid lesions reported nationally and found that the rectum was the most commonly affected site (781 lesions, 36.2%).

Carcinoid tumors are epithelial tumors that arise from endocrine cells (Kultschizky cells) in the base of gland ducts in the deep lamina propria mucosae and break through the muscularis mucosae in early stage, where they go on to grow and proliferate in the submucosal layer. Carcinoid tumors can be differentiated from other submucosal tumors with relative ease, if a hard, yellow to yellowish-white mass is present. Hasegawa *et al.* [12] studied the relationship between endoscopic findings and tumor size by examining the gross findings and surface characteristics for 88 rectal carcinoid lesions of varying sizes. They found that 26 (96.3%) of the 27 lesions smaller than 5 mm were flat or sessile, and that 2 of these had shallow depressions. Of the lesions 5 mm or larger, more tended to be semi-pedunculated, and the larger the tumor, the greater the likelihood of depressions or ulceration. These findings are largely consistent with the results of the present study.

Initially, successive studies [1,2] reported a close relationship between tumor size and biological malignancy as indicated by depth of wall invasion and lymph node and distal metastasis, suggesting that tumor size may be an indicator of malignancy. However, studies in recent years have found no relationship between tumor size and malignancy, and have revealed some cases of malignant tumors less than 10 mm in size [3–5]. It therefore makes little sense to assess the likelihood of malignancy or non-malignancy based on tumor size alone, while some attempts are being made to find a histologic indicator [7]. The aim of the present study was to try to find a new indicator of malignancy by examining the relationship between malignancy and histologic findings and growth based on endoscopic findings and tumor size previously used as indicators, focusing on lesions smaller than 10 mm and confined to the submucosal layer.

Histologically, tumors are comprised of groups of tumor cells (tumor nests) and the fibrous interstitium that surrounds those nests. Histologic tumor type is determined by classifying the arrangement of the cells within the tumor nest, and is usually based on the cell arrangement that occupies the largest area within the tumor (the structure of the dominant nest) [7,13]. For this purpose the classification by Soga *et al.* [13] is most commonly used in Japan. It classifies the tumor cell arrangement (structure of the tumor nest) into 5 types (A, B, C, D, and mixed). Iwafuchi *et al.* [7] further classified these into 9 types according to the growth stage of the cell groups. In carcinoid tumors with low-grade malignancy (classical tumors), small solid, short trabecular to trabecular, or ribbon-like nests predominates, while in highly malignant endocrine cell carcinomas, nodular or sheet-like nests is most commonly seen [14]. In the present study, examination of the structure of the dominant nest and tumor size showed that the nests of tumors smaller than 5 mm were limited to the small solid, trabecular and ribbon-like types of low-grade malignancy. This supports the findings of Kotake *et al.* [2], who found no cases of metastasis with tumors 5 mm or smaller among cases compiled from different sources. However, in a study by Shirouzu *et al.* [15], rectal wall metastasis attributed to lymph node invasion was observed in a patient with a 4-mm Soga type D lesion (corresponding to sheet-like nests in the classification by Iwafuchi *et al.*), indicating that tumor size is not an absolute indicator of tumor malignancy. The observation that lesions with surface ulceration endoscopically exhibited nodular + sheet-like nests supports the findings of other authors that such characteristics are indicative of malignancy [1,16].

We therefore investigated the relationship between degree of malignancy and growth and proliferation by evaluating the proportion of the tumor occupied by fibrous interstitium, a tumor constituent, and the composition and distribution pattern of the fibrous interstitium. Tumors smaller than 5 mm with small solid or trabecular nests had a significantly higher F/T ratio, and were formed of interstitium in which smooth muscle and collagenous fibers were mixed to form a whole. The higher F/T ratio was attributed to the fact that the tumor cell groups did not form a mass, but were instead distributed with large quantities of destroyed muscularis mucosae (smooth muscle) and collagenous fibers present in the spaces between the groups, especially when the tumor structure was at the immature stage. In tumors that have matured to some degree (5 mm or larger), the tumor cells form a solid mass in the submucosal layer, after breaking through the muscularis mucosae. At this time, the fibrous interstitium on the superior side of the tumor was comprised of smooth muscle originating from the muscularis mucosae, and the inside of the tumor was comprised of collagenous fibers that appear between the tumor nests as the tumor becomes larger. The F/T ratio was lower in lesions where the smooth muscle on the superior side of the tumor was present in greater abundance (lesions in which there was limited collagenous fibers proliferation), while higher in lesions in which the collagenous fibers inside the tumor were more abundant than smooth muscle. The above-mentioned fact suggests that in tumors that have matured to some degree, the ratio is determined by the number of collagenous fibers. Furthermore, it appears that collagenous fibers proliferate more in highly malignant lesions, since the ratio was significantly lower in tumors formed from ribbon-like nests and higher in tumors formed from nodular + sheet-like nests. Although, the ratio was high in some (3 lesions) of the tumors formed from ribbon-like nests (19 lesions) due to a large increase in the number of collagenous fibers, preoperative EUS showed that all of these lesions were deeper types in the submucosal layer, suggesting that the number of collagenous fibers in the interstitium increases further as the tumor grows and proliferates deep into the submucosal layer.

In conventional internal echo images, carcinoid tumors were recognized as homogenous low-echoic regions [17–19]. However, the advent of high-frequency devices (12 and 20 MHz) has provided better resolution, enabling detailed imaging of the interior of even smaller lesions. The fibrous components are visualized as slightly high-echoic regions, in contrast to the carcinoid tumor nests which are imaged as low-echoic regions, suggesting that it may be possible to predict the structural pattern of the inside of the tumor by focusing on the fibrous interstitium. Nomura *et al.* [20] found that many lesions in which the proportion of the tumor occupied by fibrous interstitium was either same as or greater than that occupied by the tumor cells exhibited a relatively high-echoic pattern, and that many lesions comprised of tumor nests divided by thick fibrous interstitium were non-homogenous. The findings of the present study were as follows. (1) Lesions smaller than 5 mm exhibited an internal echo image characterized by a low-echoic pattern mixed with a honeycomb-like high-echoic pattern, and were demonstrated as superficial submucosal tumors comprised of dominant nests of low-grade malignancy and large amounts of fibrous interstitium. (2) Lesions 5 mm or larger with a homogenous low-echoic internal echo image were represented as superficial submucosal tumors comprised of dominant nests of low-grade malignancy and small amounts of fibrous interstitium. (3) Lesions 5 mm or larger with an internal echo image characterized by a low-echoic pattern with multiple high-echoic dots of varying sizes contained larger amounts of fibrous interstitium than lesions with a homogenous low-echoic internal echo image. The structure of the dominant tumor nest varied from low-grade malignancy to highly malignant. These lesions were represented as deeper submucosal type tumors (4 of the 5 lesions, 80%). (4) Semipedunculated lesions 5 mm or larger could not be visualized on internal echo because of echo dumping resembled homogenous low-echoic lesions in structure and were superficial submucosal types comprised of dominant nests of low-grade malignancy and small amounts of fibrous interstitium.

A tumor size of 10 mm and proper muscle layer invasion were previously used as cut-off points when deciding whether to use endoscopic or surgical resection for rectal carcinoid tumors [16–19]. However, endoscopic resection is inadequate for deeper submucosal tumors [18], and decisions regarding additional resection should preferably be based on detailed histologic testing of resected specimens regardless of which procedure is selected. While it is known that preoperative EUS can provide useful information (e.g., tumor size, depth of invasion, presence of metastasis to perirectal lymph nodes [21]) for selecting procedures [17–19], there have been no studies on indicators of histologic malignancy. The present study proved that many of the lesions 5 mm or larger with an internal echo image characterized by a low-echoic region with multiple high-echoic dots of varying sizes were deeper types in the submucosal layer that may have been composed of highly malignant dominant nests. It may provide new information to assist in preoperative diagnosis.

### CONCLUSIONS

Following are the results of the present study aimed at finding an indicator of histological malignancy by focusing on tumor structure, dominant nest structure, and fibrous interstitium found in the spaces between tumor nests in 32 patients (33 lesions) with primary rectal carcinoid tumors.

(1) Many lesions with a histologic size of less than 5 mm were sessile (87.5%) on endoscopy and were composed of small solid or trabecular nests (75.0%). A lesion with surface ulceration endoscopically and another with advanced carcinoma-like irregular ulceration and an invasion depth of adventitia were composed of nodular + sheet-like nests.

(2) The proportion of the tumor occupied by fibrous interstitium (F/T ratio) was significantly higher in lesions smaller than 5 mm than in lesions 5 mm or larger. The F/T ratio of tumors comprised of small solid or trabecular nests was significantly higher than that of tumors composed of ribbon-like nests, and the F/T ratio of tumors comprised of nodular + sheet-like nests was also high.

(3) The fibrous interstitium was composed of part of the smooth muscle resulting from destruction of the muscularis mucosae and collagenous fibers, and was classifiable into mixed or separated based on the distribution pattern of these constituents. Lesions with mixed interstitium were immature and of low-grade malignancy. In lesions with separated interstitium, the F/T ratio was determined by the volume of collagenous fibers, and in deeper submucosal and highly malignant lesions, there was greater collagenous fiber proliferation.

(4) Lesions smaller than 5 mm on EUS had an internal echo image featuring a low-echoic tumor pattern mixed with honeycomb-like high-echoic pattern. Lesions 5 mm or larger were classifiable into: (a) those with a homogenous low-echoic internal echo image; (b) those with an internal echo image in which the low-echoic region contained multiple high-echoic dots of varying sizes; and (c) those in which the internal echo image could not be visualized due to echo dumping. It was possible to predict the tumor structure (structure of the dominant nest, F/T ratio, and fibrous interstitium distribution pattern) and depth of invasion from these EUS images, thereby providing an aid to preoperative diagnosis.

### Acknowledgments

We would like to offer our sincerest thanks to Professor Tetsu Yamaguchi and Professor Yoshihiro Sakai for assisting in completion of the manuscript by providing guidance and feedback, and Dr. Kazuya Yoshimoto (Lecturer) for his guidance throughout the study. We would also like to thank Assistant Professor Kei Takahashi of the Department of Pathology, Toho University Ohashi Hospital, and the medical staff of the Third Department of Internal Medicine at Toho University School of Medicine for their assistance in preparing the specimens.

### References

[1] Haraguchi, M., Makiyama, K., Yamakawa, M. *et al.* Six cases of rectal carcinoid treated by endoscopic polypectomy – a report of the cases and the review of Japanese literature. *Gastroenterol. Endosc.* 1988; **30**: 2612–2620 (in Japanese with English Summary).

[2] Kotake, K., Yoneyama, K., Miyata, J. *et al.* Carcinoid tumors of the rectum – report of five cases and a review of Japanese literature. *J. Jpn. Soc. Coloproctol.* 1984; **37**: 261–266 (in Japanese with English Summary).

[3] Naunheim, K.S., Zeitel, J., Kaplan, E.L. *et al.* Rectal carcinoid tumors – treatment and prognosis. *Surgery* 1983; **94**: 670–676.

[4] Tomoda, H., Furusawa, M., Hayashi, I. *et al.* A rectal carcinoid tumor of less than 1 cm in diameter with lymph node metastasis: a case report and a review of the literature. *Jpn. J. Surg.* 1990; **20**: 468–471.

[5] Iwasaki, M., Yamagiwa, K., Nakamura, K. *et al.* A case of small rectal carcinoid (7 mm in diameter) with liver metastasis. *J.J.G.S.* 1992; **25**: 1339–1343 (in Japanese with English Summary).

[6] Japanese Research Society for Cancer of the Colon and Rectum. General Rules for Clinical and Pathological Studies on Cancer of the Colon, Rectum and Anus. The 5th edition 1994 (in Japanese).

[7] Iwafuchi, M., Watanabe, H., Noda, Y. *et al.* Gastrointestinal carcinoid tumors of Japanese, Incidence and characteristics based on anatomical classification, with special reference to difference between carcinoid tumor and endocrine cell carcinoma. *Stomach Intest.* 1989; **24**: 869–882 (in Japanese with English Summary).

[8] Aibe, T. (1) A study on the structure of layers of the gastrointestinal wall visualized by means of the ultrasonic endoscope (2) The structure of layers of the oesophageal wall and the colonic wall. *Gastroenterol. Endosc.* 1984; **26**: 1465–1473 (in Japanese with English Summary).

[9] Oberndorfer, S. Karzinoid tumoren des dunndarms. *Frankfurt Z Path.* 1907; **1**: 432.

[10] Saltykow, S.L. Uber die Genese der 'Karzinoiden Tumoren' sowie der 'adenomyoma' des Darms. *Beitr. Path. Anat.* 1912; **54**: 559–594.

[11] Soga, J. Carcinoid tumors: a statistical analysis of Japanese series of 3126 reported and 1180 autopsy cases. *Acta Med. Biologica* 1994; **42**: 87–102.

[12] Hasegawa, S., Iwashita, A., Futami, K. *et al.* A clinicopathological study on rectal carcinoid with special reference to immunohistochemical factors of malignant potential. *J. Jpn. Soc. Coloproctol.* 1997; **50**: 163–176 (in Japanese with English Summary).

[13] Soga, J. and Tagawa, K. Pathologic analysis of carcinoids, histologic reevaluation of 62 cases. *Cancer* 1971; **28**: 990–998.

[14] Iwafuchi, M., Watanabe, H., Ishihara, N. *et al.* Pathology of carcinoid tumor and endocrine cell carcinoma of the digestive tract: characteristics and histogenesis. *Rinsho Shokaki Naika* 1990; **5**: 1669–1681 (in Japanese).

[15] Shirouzu, K., Isamoto, H., Kakegawa, T. *et al.* Treatment for rectal carcinoid – including D-type carcinoid. *J. Jpn. Soc. Coloproctol.* 1989; **42**: 519–525 (in Japanese with English Summary).

[16] Ishikawa, T., Ushio, K., Kusaka, K. *et al.* Carcinoid tumor of the colon: diagnosis and treatment. *Stomach Intest.* 1989; **24**: 891–902 (in Japanese with English Summary).

[17] Ootsuka, H., Shimizu, S., Iso, A. *et al.* Endoscopic ultrasonography for carcinoid tumor of the GI tract. *Gastroenterol. Endosc.* 1991; **33**: 2205–2210 (in Japanese with English Summary).

[18] Honda, Y., Mituki, O., Sekikawa, T. *et al.* Studies on determination of surgical proceduces for carcinoid tumor of the rectum according to findings of invasing depth and regional lymphnode metastasis using endoscopic ultrasonography. *J. Jpn. Soc. Coloproctol.* 1994; **47**: 106–113 (in Japanese with English Summary).

[19] Saito, K., Saito, N., Sarashima, H. *et al.* Clinical evaluation of endoscopic ultrasonography as a means for diagnosis of rectal carcinoid tumors. *Stomach Intest.* 1995; **30**: 715–721 (in Japanese with English Summary).

[20] Nomura, M., Fujita, N., Matsunaga, A. *et al.* Ultrasonographic study of rectal carcinoid tumors. *Jpn. J. Gastroenterol.* 1996; **93**: 797–805 (in Japanese with English Summary).

[21] Ishikawa, H., Imanishi, K., Tatsuta, M. *et al.* Endoscopic polypectomy in treatment of rectal carcinoid tumors. *Gastroenterol. Endosc.* 1988; **30**: 3067–3074 (in Japanese with English Summary).

